# Nurse-Implemented Music Therapy to Reduce Anxiety in Community-Dwelling Individuals with Severe Mental Illness: A Pilot Study

**DOI:** 10.3390/nursrep14020053

**Published:** 2024-03-22

**Authors:** Vanessa Ibáñez-del Valle, Vanessa Sánchez-Martínez, Josep Silva

**Affiliations:** 1 Department of Nursing, Faculty of Nursing and Podiatry, University of Valencia, 46010 Valencia, Spain; maria.v.ibanez@uv.es (V.I.-d.V.); vanessa.sanchez@uv.es (V.S.-M.); 2 Frailty and Cognitive Impairment Organized Group (FROG), University of Valencia, 46010 Valencia, Spain; 3 Valencian Research Institute for Artificial Intelligence, Universitat Politècnica de València, 46022 Valencia, Spain

**Keywords:** nursing, severe mental illness, music therapy, relaxation, anxiety

## Abstract

Anxiety is an important and recurrent problem in people with severe mental illness (SMI). The aim of this work is to measure the effectiveness of the Music Therapy nursing intervention in reducing anxiety in outpatients diagnosed with SMI (bipolar disorder and schizophrenia). The intervention was structured over five weeks (ten 1-h sessions, twice weekly). Objective measures (blood pressure, heart rate, and respiratory rate) and subjective measures (anxiety response and the subjective perception of relaxation) were taken before and after every session. Our results show that this nursing intervention entails an objective reduction of the respiratory rate ((−4.5, −0.5) breaths per minute), the heart rate ((−5.80, −2.13) bpm), and it evidences a reduction in the subjective perception of anxiety (16.08% mean reduction in state anxiety). Considering all the sessions, the subjective perception of relaxation increased 97.33% of the time. This study provides evidence that the Music Therapy intervention can effectively promote relaxation and reduce anxiety symptoms in people with SMI. This study was retrospectively registered at Clinical Trials with Protocol Identifier NCT06315049.

## 1. Introduction

There is a wide variety of definitions of music therapy, which is why it is convenient to use the generic and global definition proposed by the World Federation of Music Therapy (WFMT) in 2011, which indicates that music therapy is understood as:

“The professional use of music and its elements as an intervention in medical, educational, and everyday environments with individuals, groups, families, or communities who seek to optimize their quality of life and improve their physical, social, communicative, emotional, intellectual, and spiritual health and wellbeing. Research, practice, education, and clinical training in music therapy are based on professional standards according to cultural, social, and political contexts” [1].

Music therapy interventions can offer a range of direct benefits, including emotional expression and regulation, stress reduction and relaxation, enhanced communication skills, cognitive stimulation, social interaction, and mood enhancement. It can also offer indirect benefits, such as improved treatment engagement, diversification of treatment approaches, increased motivation and self-esteem, or cultural and individual sensitivity.

The usefulness of music therapy in different populations has been studied for years. Some relevant studies have been carried out with ICU patients [2], neonatal patients [3], stroke patients [4,5], mechanically ventilated patients [6,7], anorexia nervosa patients [8], and even with the parents of ICU patients [9]. Practically, all these studies report a positive effect of music therapy, with different levels of influence depending on the targeted population. In the same vein, various meta-reviews [10,11,12,13,14,15,16] point out that music therapy affects patients’ outcomes, and can be considered a non-pharmacological instrument to reduce anxiety and stress.

In this study, we targeted a population of severe mental illness (SMI) community-dwelling outpatients. This population often experiences severe symptoms, such as hallucinations and delusions. They may have extreme mood changes, affecting their ability to function in daily life and facing a significant decrease in social, occupational, and/or academic functioning. They find difficulties in performing daily tasks such as personal care, household management, and maintaining interpersonal relationships and may experience stigma and social isolation due to stereotypes associated with severe mental illnesses. Cognitive impairment may sometimes affect memory, attention, and other cognitive functions. For these reasons, they often require ongoing support from mental health professionals through pharmacological treatments and psychological therapies. The collaborative efforts of mental health professionals, including music therapists, can contribute to a holistic approach to mental health care.

In the specific context of mental illnesses, music therapy research has mainly focused on depression [17,18,19]. However, fewer studies exist for other mental illnesses, such as autism [20], schizophrenia [21], or bipolar disorder [22]. Many studies on this population were done with inpatients [23,24] with mild or moderate severity [25]. Nevertheless, very few studies exist for SMI outpatients.

We are not aware of any study that targeted the population evaluated in this study and assessed them with the tools used: objective measures of anxiety (SBP, DBP, HR, and RR) together with the subjective Inventario De la Ansiedad Rasgo-Estado (IDARE) test. For this reason, our results add to the existing body of knowledge on music therapy and its effects.

## 2. Objective and Research Questions

The general objective is to evaluate the effectiveness of the Nursing Interventions Classification (NIC) activity, known as Music Therapy, as a therapeutic modality complementary to psycho-pharmacological treatment for reducing anxiety in patients diagnosed with SMI. The specific research questions are:

Q1. How much do music interventions affect the objective values of vital signs (SBP, DBP, HR, and RR) in people diagnosed with SMI?
Q2. How much does the subjective perception of anxiety change after a music intervention with people diagnosed with SMI?

## 3. Materials and Methods

### 3.1. Study Design and Population

This pilot study is quasi-experimental analytical research of cross-sectional type. It is a group intervention approved by the Institutional Ethics Committee for Experimental Research with Humans of the Universitat de València (protocol code 2023-ENFPOD-2621979). It was conducted following Good Clinical Practice (GCP), and all researchers had completed GCP training before the start of the study. It was conducted with outpatients diagnosed with SMI. All patients were recruited from a community mental health center in Valencia (Spain). Fourteen patients regularly attended an occupational activities rehabilitation workshop. All of them were recruited and, after the workshop, they participated in the music therapy sessions. All the participants had been previously diagnosed with SMI (schizophrenia, bipolar disorder) by their psychiatrist. All participants were being treated with neuroleptic drugs, and many of them were also prescribed anxiolytic drugs. Every two weeks, the patients attended their psychiatrist’s office, who reviewed their treatment and conducted psychotherapy. Ten participants lived with their parents; four lived alone without familiar support. None of the participants worked. All had total permanent disability, legally granted, and therefore could not carry out any work activity.

All patients who met the inclusion/exclusion criteria and voluntarily accepted to participate were recruited: 14 patients (3 with bipolar disorder, 11 with schizophrenia) participated in the study (with a total sample of 10 group sessions and 75 individual data collection interviews). None of the patients had any previous music therapy experience. All patients followed unique treatments, and this study was an opportunity to motivate their social interaction through music and to study its impact on anxiety.

The inclusion criteria were: (i) diagnosed with SMI (schizophrenia, bipolar disorder) according to the DSM-V-TR; (ii) currently in outpatient psychiatric treatment in the corresponding mental health unit; (iii) acceptance to enter the study (informed consent); if legally incapacitated, authorization from the patient’s legal guardian; (iv) ability to understand the questions in anxiety questionnaires; and (v) between 35 and 50 years of age, to focus the study on a specific population (young adulthood above 35 years and middle adulthood) and to narrow the age range so that common recall of musical memories could be shared. The exclusion criteria were: (a) suffering from a dual pathology (diagnosis of mental illness and, at the same time, substance abuse) and in the dependency phase; (b) affected by some type of degenerative disease (dementia, Alzheimer’s, etc.); (c) presence of positive psychotic symptoms or behavioral disorganization susceptible to admission in the mental health unit; and (d) deafness. The patients’ psychiatrists determined the cognitive competency of the patients and decided on the convenience of their participation.

### 3.2. Music Therapy Sessions

The design uses a one-group intervention with pre- and post-tests made before and after each of the 10 sessions. The intervention was organized into ten 1-h music therapy sessions at the outpatient level twice weekly. Patients’ adherence to the music therapy sessions was 54%, which was more or less stable (standard deviation 11%). The average attendance was 8 people. The minimum was 5, and the maximum was 10.

The group music therapy sessions were conducted according to the Benenzon model of music therapy [26]. This model of music therapy focuses on music/sound interaction within a non-verbal framework. Its main goal is to enhance interpersonal communication and facilitate emotional expression, improving quality of life and well-being [27]. This model uses bodily–sound-nonverbal elements to develop, process, analyze, and strengthen a bond or relationship between the music therapist and the patient (or group of patients) to achieve the patient’s well-being.

A pre-intervention interview was carried out. The nurse explained the intervention individually, and each participant signed an authorization to record a video of the sessions and to give the informed consent. The musical and non-musical preferences of each participant were collected following Poch’s (1999) model, which includes the completion of the sound-music file (SMF) questionnaire [28]. SMF is a questionnaire used to find an individual’s musical preferences and identify songs or music that produce good or bad memories and feelings. The nurse created a Music Therapeutic History of each participant with this information. It contained a record where music memories, musical preferences (songs, singers, groups, instruments), dancing preferences, sounds, and noise that the participant liked or disliked were registered. The information provided by the families of the participants was also considered. This was carried out following an interview model adapted from [29]. The selected music, tailored to patients’ assessment and preferences, was strategically designed with reference to all this information for each session. This was particularly useful in promoting relaxation with specific songs for those participants who, at some point, felt overstimulated by one exercise (e.g., body activation, percussion, etc.). The participant was invited to stop the exercise and was attended by the nurse assistant. The other participants continued the exercise with the nurse. The participant recovered quickly and returned to the group with the next exercise. The nurse interviewed the participant and registered the songs, instruments, rhythms, and exercises that affected him, to avoid these in the design of the following sessions.

Before starting the intervention sessions, a first assessment (pilot) session was held to let patients know the session’s dynamics and assess their needs. The same nurse directed all sessions with the help of an assistant. The nurse holds a master’s degree in mental health and a master’s degree in music therapy. She had 18 years of experience as a mental health specialist nurse and 12 years as a music therapist.

The assistant controlled the music (songs, volume, etc.), directed by the music therapist. All sessions were carried out in the same music-dedicated room in the mental health center, with 5.1 speakers surrounding the group, sitting in a circle (or standing according to the activity) because this favors eye contact, interaction, participation, and increases the sense of belonging. The music equipment was situated two meters away from the group. The room ensured privacy and low ambient sound.

The proposed activities allowed significant interaction between participants. The structure of the session is shown in Table 1.

Ten music therapy sessions were carried out with a frequency of two times/week, so the whole intervention lasted five weeks. The approximate duration of each session was 60 min. Before and after each session, all participants completed the IDARE test, and their SBP, DBP, HR, and RR were registered. After each session, they were also asked whether they felt less relaxed, the same, or more relaxed than before the session.

The material resources used during the sessions were musical instruments: djembe, pandero, woodblock, claves, maracas, headless tambourine, agogô, triangle, sistrum, güiro, rain-stick, balafon, guitar, and standing bell, as well as audio equipment, video recorder, and a selected discography in MP3 format according to the participants’ musical preferences.

### 3.3. Evaluation of the Subjective Perception of Relaxation and Anxiety

The subjective perception of anxiety was measured with a validated tool: *The State-Trait Anxiety Inventory* (STAI) [30]. The STAI is commonly used to obtain a meaningful measure of state anxiety (SA) and trait anxiety (TA). It is also often used in clinical settings to diagnose anxiety and distinguish it from depressive syndromes. In this study, we used IDARE [31], the Spanish version of STAI, published by Spielberger et al. with the help of psychologists from ten Latin-American countries. IDARE has also been validated with a Spanish population [32,33]. IDARE is a form with 20 items to address trait anxiety and 20 items to address state anxiety. All items are scored from 1 to 4: the higher the score, the greater the anxiety. Data were collected by trained personnel via face-to-face evaluations.

At the end of each session, all participants were asked about their overall subjective perception of relaxation (SPR) compared to their state before the session:

“After this session, how do you feel?
0. Less relaxed than before.
1. The same as before.
2. More relaxed than before.”

### 3.4. Evaluation of Vital Signs

A nurse registered four vital signs before and after every music therapy session: SBP, DBP, HR, and RR.

### 3.5. Correlation between Objective and Subjective Measures

A bivariate correlation was performed between all objective and subjective measurements of relaxation (both at the beginning and at the end of the sessions). The results can provide information about what objective variables are a better indicator of the subjective perception of anxiety.

### 3.6. Statistical Analysis

We used SPSS 20.0 to perform all statistical analyses. The results are presented as the mean ± standard deviation. We also performed the Shapiro–Wilk test to check whether the data followed a normal distribution. All studied variables followed a normal distribution, except SPR and the differences found before and after the music therapy sessions in the values of HR, RR, and state–trait anxiety. To study these variables with a non-normal distribution, we used non-parametric statistical analysis tools. The Kruskal–Wallis test was used to analyze group differences. In contrast, bivariate correlations between variables were evaluated using the Spearman correlation test and multivariable-adjusted statistical analyses. To compare the pre- and post-intervention results, we used the paired *t*-test for normally distributed variables and the Wilcoxon signed-rank test for non-normally distributed variables. *p* < 0.05 values were considered to be statistically significant. We used Cohen’s *d* for parametric tests to study the effect size and Hedges’ *g* for non-parametric tests. Because several variables were examined, thus making multiple comparisons, we applied a multiple testing correction using the Bonferroni method (free of dependence and distributional assumptions) with an alpha adjustment of 7 (SBP, DBP, HR, RR, SA, TA, SPR). We did not observe a significant difference in response based on the psychiatric diagnosis (schizophrenia or bipolar disorder).

## 4. Results

### 4.1. Evaluation of Anxiety with Objective Measurements

The mean, median, and interquartile range (IQR) values obtained by measuring the vital signs (SBP, DBP, HR, and RR) before and after the music therapy sessions are shown in the upper part of Table 2. This table is complemented by Table 3, which shows the percentage of users whose studied variables increased, stayed the same, or decreased (showing the mean ± the standard deviation considering all sessions). The *t*-tests results did not show a significant difference between the measures taken pre- and post-intervention for systolic (*p* < 0.728) and diastolic (*p* < 0.961) blood pressures. In contrast, the Wilcoxon signed-rank test results showed a significant difference between the measures taken pre- and post-intervention for the heart (*p* < 0.001) and respiratory (*p* < 0.001) rates.

*Blood Pressure*. This was measured with an electronic blood pressure monitor. The mean SBP decreased (by −7.13 to −2.25 mmHg) in 40% of the sessions, and increased (by +0.40 to +4.25 mmHg) in 60% of the sessions. As for the DBP, it decreased (by −7.71 to −0.25 mmHg) in 60% of the sessions and increased (by +0.50 to +5.83 mmHg) in 40% of the sessions.
*Heart Rate*. This was measured with an electronic heart rate monitor. The mean HR decreased (by −5.80 to −2.13 bpm) in 90% of the sessions and increased (+1.3 bpm) in 10%.
*Respiratory Rate*. This was measured by counting the number of breaths for one minute. The RR did not increase in individual measurements considering all sessions. The mean RR decreased (by −4.5 to −0.5 breaths per minute) in 100% of the music therapy sessions performed.

**Table 2:** Objective and subjective evaluation of anxiety and relaxation (N = 14).

		Age Range	35–50	Gender	29% F, 71% M
		Range	Mean ± SD	Median	IQR
Objective Measurements	Initial Systolic Blood Pressure	[90, 149]	110.15 ± 11.11	108	12
	Final Systolic Blood Pressure	[93, 139]	110.59 ± 9.72	111	14
	Difference Systolic Blood Pressure (final-initial)	[−26, 24]	0.44 ± 10.89	0	13
	Initial Diastolic Blood Pressure	[57, 101]	74.39 ± 8.72	73	13
	Final Diastolic Blood Pressure	[54, 94]	74.35 ± 8.55	73	12
	Difference Diastolic Blood Pressure (final-initial)	[−15, 20]	−0.04 ± 6.86	−1	7
	Initial Heart Rate	[46–120]	82.09 ± 15.01	82	15
	Final Heart Rate	[47–109]	78.84 ± 13.27	79	14
	Difference in Heart Rate (final-initial)	[−20, 13]	−3.25 ± 5.50	−2	6
	Initial Respiratory Rate	[16, 36]	23.52 ± 4.55	24	4
	Final Respiratory Rate	[16, 32]	21.23 ± 3.40	20	4
	Difference in Respiratory Rate (final-initial)	[−12, 0]	−2.29 ± 1.17	−2	4
Subjective Measurements	Initial State Anxiety	[26, 78]	42.49 ± 12.22	40	18
	Final State Anxiety	[21, 76]	35.92 ± 9.16	35	11
	Difference in State Anxiety (final-initial)	[−41, 7]	−6.57 ± 8.57	−5	10
	Initial Trait Anxiety	[23, 76]	46.96 ± 11.23	46	14
	Final Trait Anxiety	[20, 71]	43.64 ± 10.61	43	12
	Difference in Trait Anxiety (final-initial)	[−33, 18]	−3.32 ± 7.28	−2	8
	Final Relaxation Perception (0-Less; 1-Equal; 2-More) than	[0, 2]	1.96 ± 0.26	1	0

**Table 3:** Number of users that experienced a variation in the quantitative variables studied.

	SBP	DBP	HR	RR	SA	TA	SPR
↑	50.05 ± 25.53%	39.78 ± 26.51%	18.66 ± 14.70%	0.00 ± 0.00%	13.40 ± 12.23%	27.75 ± 13.22%	97.57 ± 5.22%
=	6.17 ± 8.23%	8.53 ± 7.77%	6.58 ± 8.68%	46.71 ± 22.50%	9.11 ± 13.05%	7.08 ± 9.63%	1.00 ± 3.16%
↓	43.79 ± 19.17%	51.69 ± 25.38%	74.75 ± 18.82%	53.29 ± 22.50%	77.49 ± 14.45%	65.17 ± 15.23%	1.43 ± 4.52%

Each cell contains the percentage of users (mean ± SD considering all sessions) whose studied variables increased (↑), stayed the same (=), or decreased (↓) between the start and the end of the session. SBP: systolic blood pressure, DBP: diastolic blood pressure, HR: heart rate, RR: respiratory rate, SA: state anxiety, TA: trait anxiety, SPR: subjective perception of relaxation.

### 4.2. Evaluation of Anxiety with Subjective Measurements

We first measured the internal consistency of the IDARE anxiety measurements with Cronbach’s alpha and obtained α = 0.893. This shows a high intercorrelation among the tests and indicates internal consistency and reliability of the measure in our sample. The mean values obtained with the IDARE’s state–trait anxiety measures and the overall anxiety perception before and after the music therapy sessions are given in the lower part of Table 2. The *t*-tests showed a significant difference between the measures taken pre- and post-intervention for trait anxiety (*p* < 0.001). Similarly, the Wilcoxon signed-rank test showed a significant difference between the measures taken pre- and post-intervention for state anxiety (*p* < 0.001).

*State Anxiety (SA)*. The mean decreased (by −13.80 to -2.50) in all sessions. Considering all measurements, the music therapy sessions reduced SA by −6.89 points over a maximum of 80. The mean SA before the sessions was 42.86 (medium level), and the mean reduction was 16.08%.
*Trait Anxiety (TA)*. The mean TA decreased (by −1.25 to −9.75) in all sessions but one, due to the presence of an outlier (one participant increased TA by 18 points). Excluding the outlier, all sessions reduced TA by a mean of −3.40 points over a maximum of 80. Considering that the mean TA before the sessions was 46.76 (medium level), the mean reduction was 7.27%.
*Subjective Perception of Relaxation (SPR)*. Out of 75 answers, 73 gave option 2. Only one response was 0, and only one answer was 1. Therefore, in 97.33% of instances, the subjective perception of relaxation increased after the music therapy session.

### 4.3. Correlation between Objective and Subjective Evaluation of Anxiety

As expected, there was a significant correlation between the scores registered at the beginning and end of the sessions for SBP (Hedges’ g −0.040), DBP (Cohen’s d 0.006), HR (Cohen’s d 0.591), RR (Hedges’ g 0.901), SA (Hedges’ g 0.759), and TA (Cohen’s d 0.456). When we studied bivariate correlations among all of the objective variables with state anxiety, we found that only RR was correlated (rho = −0.39 *p* < 0.01, Spearman correlation test; Hedges’ g 0.451) at the beginning of the sessions and at the end of the sessions (rho = −0.40 *p* < 0.01, Spearman correlation test).

This indicates that the respiratory rate is a better indicator of state anxiety in this population. The other correlations were weaker, and they were only found at the beginning of the sessions: initial DBP with initial SA (rho = −0.34 *p* < 0.01, Spearman correlation test). We also found that initial SBP with initial SA (rho = −0.27 *p* < 0.05, Spearman correlation test) and initial HR with initial SA (rho = −0.28 *p* < 0.05, Spearman correlation test) correlated with a confidence level of 5%. Still, these correlations were not considered according to the Bonferroni alpha adjustment. The respiratory rate is the only variable that never increased (for any user in any session).

As regards the subjective perception of relaxation, it was found to (inversely) correlate only with the final TA (rho = −0.26 *p* < 0.01, Spearman correlation test) and with the difference in the TA (rho = −0.26 *p* < 0.01, Spearman correlation test). No significant correlation was found between the subjective perception of relaxation and SA or the other variables under study.

### 4.4. Influence of Music Therapy Sessions on Anxiety

We measured the influence of the sessions by separately analyzing each day to determine whether the session itself (probably including, for example, the specific music selected, the activities carried out, the therapist’s planning, etc.) had a significant influence on anxiety reduction. No significant correlation was observed between the different sessions and the values of any variable at the beginning or end of the sessions. However, we found correlations between the sessions and the differences between the values observed at the beginning and end for different variables.

We found a significant inverse correlation between the sessions and the differences between the initial and final values for RR (rho = −0.23 *p* < 0.01, Spearman correlation test), DBP (rho = −0.24 *p* < 0.01, Spearman correlation test), and for SA (rho = −0.24 *p* < 0.01, Spearman correlation test). This means that the configuration of the sessions could influence anxiety reduction.

In summary, we have the following results:

- Music therapy can reduce the respiratory rate ((−4.5, −0.5) bpm).
- Music therapy can reduce the heart rate (by −5.80 to −2.13 bpm).
- Music therapy can reduce state anxiety (IDARE test: 16.08% mean reduction).
- Music therapy increased the subjective perception of relaxation 97.33% of the time.

## 5. Discussion

This work targeted a specific population for which anxiety is common: outpatients diagnosed with SMI. Similar studies to are those by Hayashi et al. [24] and Degli Stefani and Biasutti [22]. These focused on measuring the effects of music therapy on the total quantity and dosages of drugs taken. Doses of neuroleptics decreased significantly in the experimental group and increased substantially in the control group. Doses of antidepressants did not change significantly in the experimental group but increased considerably in the control group. In contrast, benzodiazepines and mood stabilizers did not significantly change in either group. The main difference with Hayashi et al. [24] is that they considered inpatients, whereas our study targeted outpatients; while the main difference with Degli Stefani and Biasutti [22] lies in the variables evaluated: they measured the number of drugs taken, and we measured the effects on anxiety along with other objective and subjective measurements. Furthermore, their study assessed the data before and after the entire intervention, while we assessed the patients before and after every session.

Some papers exist that studied the effects of music therapy on reducing anxiety in people with SMI (see [34,35,36], among others). However, only in one [34] were the participants community-dwelling individuals. Other studies were carried out in hospitals. The study by Grocke et al. [34] used different metrics: the WHOQOLBREF Quality of Life (QoL) Scale and the Social Interaction Anxiety Scale (SIAS).

Aalbers et al. [37] argue that music therapy can help modulate mood and emotions. Group intervention favors social interaction, generating synergies of collective enjoyment that favor social support, reducing anxiety. A social support network helps to reduce stress. For this reason, the intervention in this work focuses on the group, in order to cover one of the primary deficiencies of participants with SMI: social interaction.

*Blood Pressure*. The statistical analysis revealed that the average SBP rose (by +0.40 to +4.25 mmHg) in 60% of the sessions. This result contrasts with other investigations. For instance, a meta-review [38] reported that music improved systolic blood pressure (−6.58 ± 2.85). Similarly, Montinari et al. [39] concluded that music therapy helped improve arterial pressure. Our data analysis revealed that some sessions gave different (incomparable) results in both SBP and DBP and practically all the variables studied. In some sessions, most patients’ SBP or DBP increased; in others, most patients’ SBP or DBP decreased. We reviewed the planning of the sessions and, even though the general objective was relaxation, we found that the configuration of the music therapy session (kind of music selected, tempo, individual and group activities carried out, etc.) was a potent modulator of the effects caused by music therapy. This gives the therapist a significant role (and responsibility). The study of different configurations is outside the objectives of this work and should be investigated in longitudinal studies. We performed the non-parametric Kruskal–Wallis test to study the differences between sessions in the variables studied, finding no differences concerning any of the variables.

*Heart Rate*. Our result stating that the mean HR decreased (by −5.80 to −2.13 bpm) in 90% of the sessions was expected, because the last part of the session was always relaxation (see Section 6 in Table 1). Koelsch and Jäncke [40] showed that HR is higher in response to exciting music than to calming music.

*Respiratory Rate*. Our results concerning the reduction of RR in 100% of the sessions have not been seen before. We are not aware of any other similar study on subjects diagnosed with SMI. There are, however, studies on different populations, such as children and adolescents [41], chemotherapy patients [42] and even cats [43]. As with HR, we also expected an RR reduction due to the final relaxation stage of the sessions. Interestingly, our results show that the same music therapy session had more influence on RR than on HR in our targeted population.

*IDARE Test*. It is worth noting that the participants in this study started (pre-intervention) with high anxiety levels, above the mean in both SA and TA. The reduction in subjective anxiety (according to the IDARE test) achieved in our experiment is significant, and the level of anxiety was reduced in all sessions. This reduction had been previously observed in other studies with different target populations. For instance, in a study by Florit and Mieres [44], the level of state anxiety also fell, but to a lesser extent than in our intervention. In that study, the initial SA was 27.25 (low level), and the final SA was 24 (low level), so a decrease of 3.25 points was reached regarding the values before the intervention. Therefore, it was more effective to diminish SA with music therapy than with their alternative program consisting of three modules (psycho-education in anxiety, training in abdominal breathing at two times, and reduced Jacobson’s training in muscular relaxation). In contrast, TA was reduced more in the study by Florit and Mieres than in our research: TA = 34.25 (medium level) before the intervention and TA = 25.75 (low level) at the end; hence a decrease of 8.5 points was achieved regarding the values before the intervention.

In our case, it is interesting to observe that the TA was quite variable (weekly) in this population even though TA is assumed to be stable over long periods; but in this case, perhaps influenced by the SA itself or by the patient’s mental illness, the inter-weekly variation was significant. The mean reduction of TA at the beginning and end of the session was 7.27%. We studied this difference because TA is supposed to remain unchanged after the session. This was due to slight variations in some items. The TA scale ranges between 1 and 4. Because IDARE is a subjective test, we think that happiness and euphoria after the session could slightly influence the patients’ answers, and in particular doubts before the session between answering, e.g., 2 or 3 to “I am content”, producing 2 before the session and 3 after the session. These slight variations affected practically all patients, but the overall impact was small (−3.40/80). This idea aligns with a study [34] that shows that “the use of music in the nurse’s activities in mental health represents a re-signification of nursing care and favors the user’s subjectivity”.

Subjective Perception of Relaxation. The music therapy sessions increased the subjective perception of relaxation 97.33% of the time. This number should be taken with some caution, because the population (SMI patients) tends to inadequately perceive the levels of anxiety they experience, perceiving less anxiety than they actually experience. This was observed, for example, in the study by Florit and Mieres [44], where the subjective perception of anxiety before and after the intervention was evaluated. In that study, the levels of anxiety that patients perceived or considered they were experiencing were lower than the levels of anxiety reflected by the questionnaires they filled in, both pre- and post-intervention.

Some conclusions can be drawn from the assessment of the data collected: (i) the respiratory rate is a good indicator of state anxiety in this population because it correlated with SA at the beginning and end of the sessions. However, we cannot conclude that there is a causative relationship. (ii) A significant inverse correlation was found between the specific session considered and the changes experienced in RR and DBP, but also with the reduction in SA achieved; all indicate that the configuration of the music therapy session (the specific music selected, the activities carried out, the planning by the therapist, etc.) had a significant influence on reducing anxiety.

Interestingly, the objective measurements of relaxation correlated more with the subjective measures at the beginning of the sessions than at the end. In particular, initial values for SBP, DBP, HR, and RR correlated with the initial value for SA, but only final RR correlated with final SA. Several reasons may explain this fact, whose exploration is left for further longitudinal studies. Our interpretation is that the sense of well-being induced by the music therapy session may influence patients’ subjective perception of relaxation at the end of the session. This should be considered in future studies.

## 6. Limitations

The sample of this study is not random. It comprises all patients who attended a specific mental health center daily. Therefore, all were a population from a particular region, and therefore, the findings cannot be generalized. All patients were diagnosed with an SMI. All were patients with schizophrenia or bipolar disorder. Thus, this study is limited to these two illnesses; no conclusion can be extracted for other conditions.

This study was retrospectively registered at clinicaltrials.gov. The failure to pre-register the study introduced a substantial risk of selective outcome reporting.

Another limitation is that the measurement of vital signs after the music intervention was not performed on all participants simultaneously. The constants were taken by a single nurse, who could not measure all participants simultaneously. Some participants had their constants taken immediately at the end of the music therapy session, while others had to wait for a short period for their turn to be taken and their vital signs measured.

One of the main external factors affecting the experiment was absenteeism (the adherence was 54%). To control this possible problem, before every session the nurse and the assistant telephoned all the participants to remind them that there was a music therapy session.

## 7. Conclusions

This study provides limited evidence (with different objective measures) that suggests that the “music therapy” nursing intervention may have therapeutic benefits. The identified correlations showed that this music intervention could be a complementary therapeutic modality to psycho-pharmacological treatment in promoting relaxation and reducing anxiety in patients with SMI. The study provides quantitative data on relaxation and anxiety useful for nursing practice. It is important to highlight the challenges and benefits of performing this technique: the main challenge is to achieve regular attendance to the sessions. Even though the patients enjoy this activity, the negative symptoms of their mental illness make their participation difficult. For this reason, we recommend calling them before the sessions to remind them to attend and make them feel that they are important to the other partners. With regards to the benefits, apart from the physical and mental benefits of the sessions, the group intervention could enhance social interaction in some people who usually have social isolation, as a result of the negative symptoms of their mental illness. Moreover, this periodic activity allows the nurse to prematurely identify and manage a relapse or pharmacological treatment discontinuation.

## Figures and Tables

**Table 1:** Structure of the music therapy sessions.

Welcome and greeting (5 min)
♦ Brief verbal dialogue to welcome and positively reinforce patient attendance at the session
♦ Welcome song
2. Personal guidance (5 min)
♦ Presentation of the participants with musical activity
3. Activation and body expression (9 min)
♦ Body activation
♦ Body expression games and exercises
4. Central activity (15 min)
♦ Work with selected songs
♦ Musical dialogue
♦ Imitation of rhythms
♦ Instrumental playbacks
♦ Song with music-gram
♦ Auditive discrimination
5. The moment for the leading role of a member of the group (6 min)
♦ Instrumental accompaniment of edited music chosen by a member of the group
6. Relaxation (20 min)
♦ Listening to edited music with verbal and/or body responses
♦ Musical listening with a guided visualization of landscapes
♦ Sound bath with Tibetan bowl

## Data Availability

The data presented in this study are available on request from the corresponding author. The data are not publicly available due to privacy restrictions.

## Public Involvement Statement

There was no public involvement in any aspect of this research.

## Guidelines and Standards Statement

This manuscript was drafted based on the guidelines for non-randomized controlled trials (TREND).
